# Induction of internal circadian desynchrony by misaligning *zeitgebers*

**DOI:** 10.1038/s41598-022-05624-x

**Published:** 2022-01-31

**Authors:** Isabel Heyde, Henrik Oster

**Affiliations:** Institute of Neurobiology, University of Lübeck, CBBM (House 66), Marie Curie Street, 23562 Lübeck, Germany

**Keywords:** Circadian mechanisms, Circadian regulation

## Abstract

24-h rhythms in physiology and behaviour are orchestrated by an endogenous circadian clock system. In mammals, these clocks are hierarchically organized with a master pacemaker residing in the hypothalamic suprachiasmatic nucleus (SCN). External time signals—so-called *zeitgebers—*align internal with geophysical time. During shift work, *zeitgeber* input conflicting with internal time induces circadian desynchrony which, in turn, promotes metabolic and psychiatric disorders. However, little is known about how internal desynchrony is expressed at the molecular level under chronodisruptive environmental conditions. We here investigated the effects of *zeitgeber* misalignment on circadian molecular organisation by combining 28-h light–dark (LD-28) cycles with either 24-h (FF-24) or 28-h feeding-fasting (FF-28) regimes in mice. We found that FF cycles showed strong effects on peripheral clocks, while having little effect on centrally coordinated activity rhythms. Systemic, *i.e.*, across-tissue internal circadian desynchrony was profoundly induced within four days in LD-28/FF-24, while phase coherence between tissue clocks was maintained to a higher degree under LD-28/FF-28 conditions. In contrast, temporal coordination of clock gene activity across tissues was reduced under LD-28/FF-28 conditions compared to LD-28/FF-24. These results indicate that timed food intake may improve internal synchrony under disruptive *zeitgeber* conditions but may, at the same time, weaken clock function at the tissue level.

## Introduction

Life on Earth is characterised by recurrent changes in environmental conditions. The circadian clock system evolved to anticipate daily recurring events, *e.g.*, light–dark cycles or changes in food availability or the presence of predators. In mammals, molecular clocks are present in nearly all cells, forming a network, which must be synchronised to generate coherent rhythms in behaviour and physiology^1,2^. This circadian clock network is organised in a hierarchical manner with a central pacemaker residing in the hypothalamic suprachiasmatic nucleus (SCN)^3–5^. At the molecular level, circadian clocks are composed of interlocked transcriptional-translational feedback loops. In the core loop, the transcription factors brain and muscle aryl hydrocarbon receptor nuclear translocator-like protein 1 (BMAL1 or ARNTL) and circadian locomotor output cycle kaput (CLOCK) regulate rhythmic expression of *Period* (*Per1-3*) and *Cryptochrome* (*Cry1/2*) and other clock-controlled genes, *e.g., D-site albumin promotor-binding protein* (*Dbp)* or *Reverse-erythroblastosis virus α* and *β* (*Rev-Erbα/β* or *Nr1d1/Nr1d2*).

So called *zeitgeber*s, external time cues, entrain the circadian system to align with geophysical time. Light is the most potent *zeitgeber* for the mammalian circadian clock system. The SCN receives photic signals from the retina via the retinohypothalamic tract^6^ and resets subordinate tissue clocks throughout the brain and peripheral tissues. SCN-mediated routes of synchronisation comprise innervation, humoral signals, and regulation of behavioural outputs^7–11^. Time of food intake strongly impacts peripheral tissue clocks. Temporal restriction of food access to the rest phase (*i.e.*, night in humans and day in nocturnal rodents) can uncouple peripheral tissue clocks from the SCN within a week^8,9^. Such internal desynchronization of the clock network by misaligned *zeitgeber* input is suggested to promote the development of shift work associated diseases, *e.g.,* obesity, type-2 diabetes, cardiovascular disorders, and major depression^12–16^, though little is known about the molecular underpinnings of such phenomena^16^. The temporal coordination of *zeitgeber* input has been proposed as a potential tool to prevent or treat shift work-associated disorders^17–19^. Therefore, dissecting the impact of different *zeitgeber*s on clock function under chronodisruptive conditions may help to devise preventive strategies for pathologies promoted by chronodisruptive environmental conditions.

Here, we investigate the effect of acute *zeitgeber* misalignment on internal rhythm coherence at systemic and tissue levels. Our results suggest a differential role of feeding rhythms in the regulation of internal (mis-)alignment at systemic and tissue levels.

## Results

### Locomotor activity period is largely independent of feeding time

**Figure 1:**
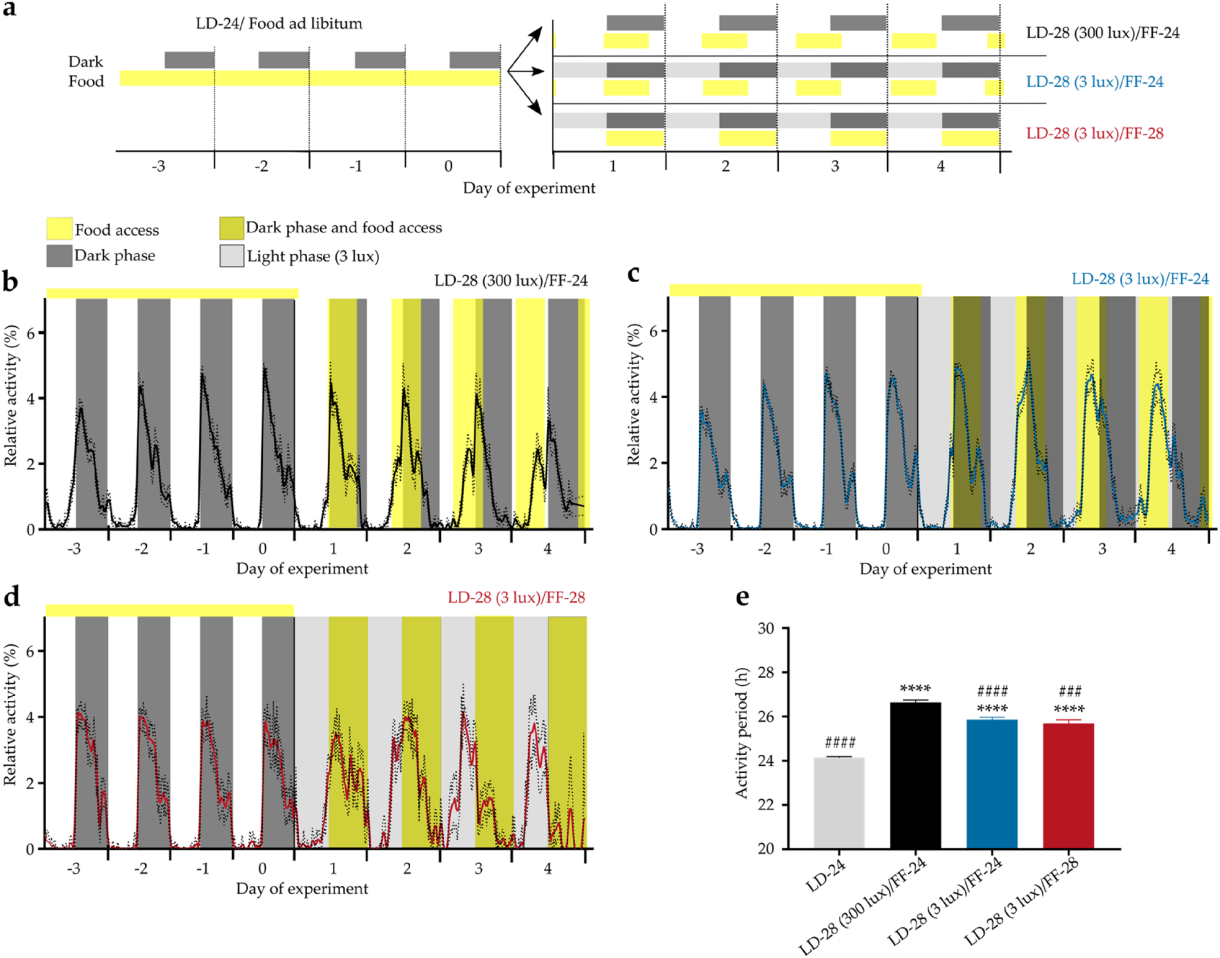
Locomotor activity rhythms under conflicting *zeitgeber* conditions. (**a**) Experimental paradigm. Normalized running-wheel activity profiles in LD-24 (experimental days -3 to 0) and in LD-28 conditions (experimental days 1 to 4) under (**b**) LD-28/FF-24 (300 lx, black, n = 39–40), (**c**) LD-28/FF-24 (3 lx, blue, n = 59–66) and (**d**) LD-28/FF-28 (3 lx, red, n = 8–15) conditions. (**e**) Calculated activity periods from activity onsets (LD-24, n = 127; LD-28 (300 lx)/FF-24, n = 55; LD-28 (3 lx)/FF-24, n = 64, LD-28 (3 lx)/FF-28, n = 10). Dark phases and food access times are indicated in dark grey and yellow shadings, respectively. Light grey shading in (**a**, **c**, **d**) indicates experimental light phase (3 lx). Data are shown as means ± SEM. Dotted lines (**b**–**d**) and error bars (**e**) indicate SEMs. **** *p* < 0.0001 *vs.* LD-24, ^###^ *p* < 0.001 and #### *p* < 0.0001 *vs.* LD-28 (300 lx)/FF-24; one-way ANOVA with Tukey's post-test.

After entrainment to a standard 12-h light: 12-h dark cycle with food ad libitum (LD-24) mice were transferred to a 28-h LD cycle (14 h light (300 or 3 lx): 14 h dark; LD-28) combined with either a 24- (12 h feeding: 12 h fasting; FF-24—with food access in the 12 h of darkness during the preceding LD-24 cycle) or a 28-h FF regimen (14 h feeding: 14 h fasting; FF-28—with food access coinciding with the 14-h dark phase; Fig. 1a). Of note, on the fourth LD-28 cycle, the light phase was 12 h phase-shifted compared to the initial LD-24 cycle. In LD-24, mice were mainly active during the dark phases as expected for nocturnal animals (Fig. 1b–d; days -3 to 0). Under LD-28 conditions, mice did neither entrain to the LD (period length (τ) = 28 h) nor to the FF cycle (τ = 24 or 28 h). Instead, all animals showed a stable intermediate activity period (Figs. 1e and S1a–c). Under LD-28/FF-24 conditions, an increase in light intensity positively affected τ (26.64 ± 0.11 h at 300 lx *vs.* 25.86 ± 0.11 h at 3 lx; Fig. 1b, c, e). In contrast, locomotor activity period was largely insensitive to the feeding regimen (25.86 ± 0.11 h under LD-28/FF-24 *vs.* 25.69 ± 0.18 h under LD-28/FF-28; Fig. 1c–e).

Together, mice showed lengthened locomotor activity period without entrainment to LD-28 conditions. Lengthening of the locomotor activity period was independent of the imposed feeding rhythm but sensitive to changes in LD light intensity.

### Phase shifts in peripheral clock gene expression depend on feeding regimes

**Figure 2:**
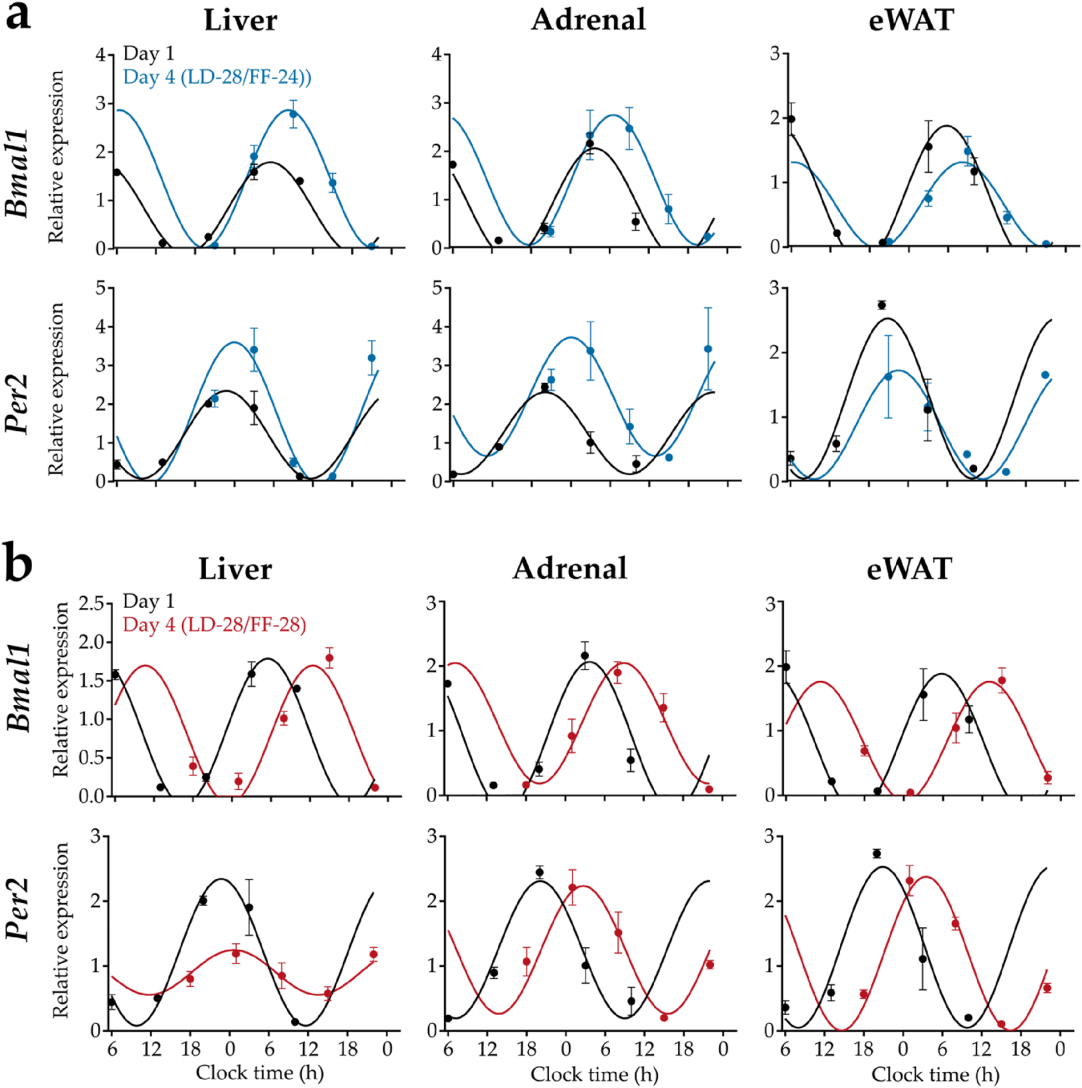
Clock gene expression profiles on the first and the fourth day under LD-28/FF-24 and LD-28/FF-28 conditions. Diurnal mRNA expression profiles over 28 h for *Bmal1* (upper panel) and *Per2* (lower panel) on days 1 (black) and 4 (blue, red) under (**a**) LD-28/FF-24 and (**b**) LD-28/FF-28 conditions in liver, adrenal, and epididymal white adipose tissue (eWAT; left to right). Data are shown as means ± SEM; n = 3–5 animals per time point. Fitted curves are sine waves with a wavelength of 25.8 h.

Clock gene mRNA rhythms were measured on the first and fourth day of the LD-28 cycle to investigate the interactive effects of LD and FF cycle period on molecular clock resetting in liver, adrenal gland, and epididymal white adipose tissue (eWAT). On day 1, gene expression rhythms for *Bmal1*, *Per2, Dbp*, *Cry1, Nr1d, Per3* and *Nr1d2* (Fig. 2a, b, Figure S2a, b) were phased as described in previous publications^20–22^.

**Figure 3:**
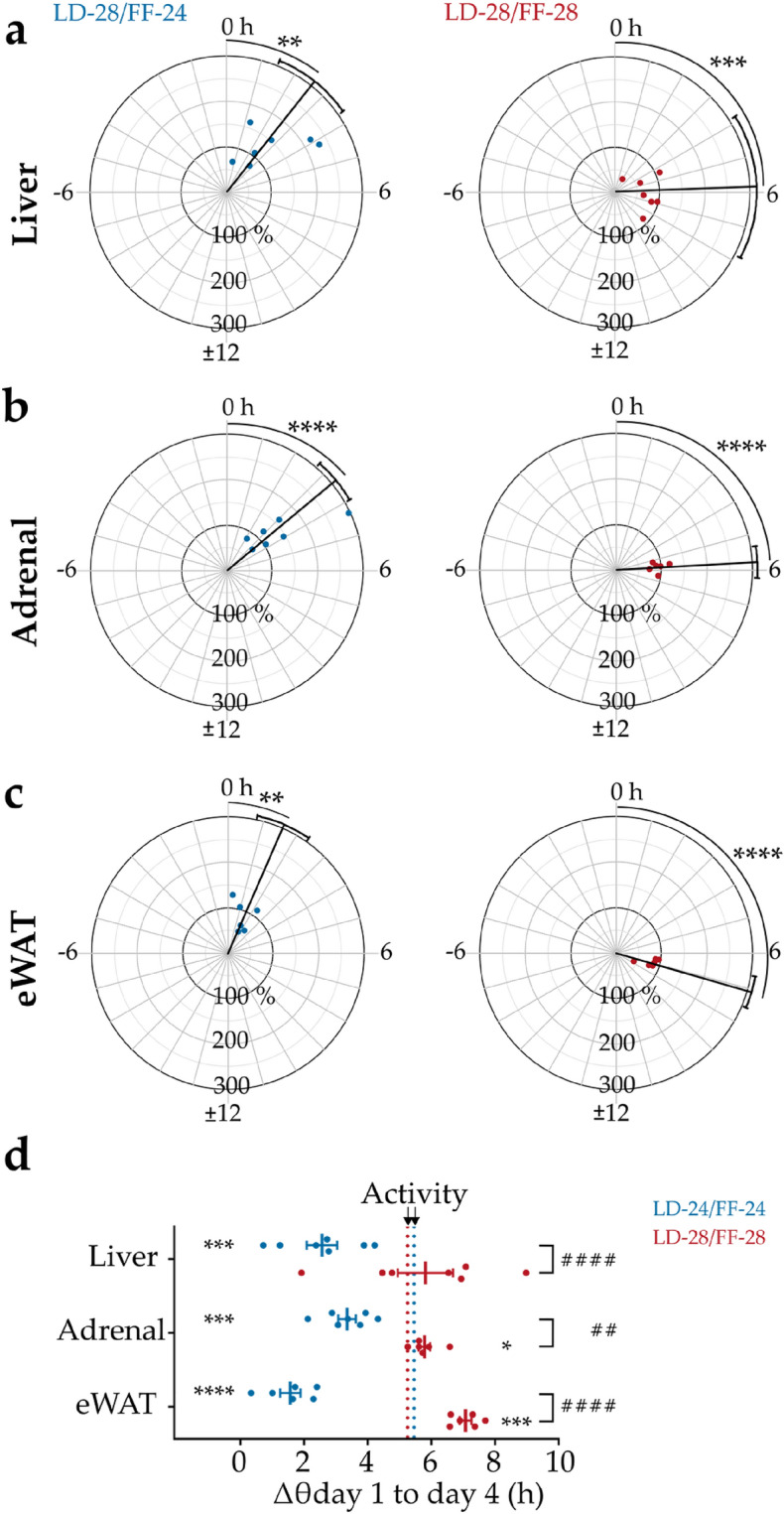
Phase and amplitude effects of clock gene expression rhythms between day 1 and 4 under LD-28/FF-24 and LD-28/FF-28 conditions. Phase shifts of clock gene peak expression calculated from sine fit maxima for LD-28/FF-24 (blue) and LD-28/FF-28 (red) conditions in (**a**) liver, (**b**) adrenal, and (**c**) eWAT. Phase shifts (in hours) and normalised amplitudes relative to baseline (in % of baseline) are shown on radial and axial axes, respectively. Black lines indicate mean phase shifts ± 95% confidence intervals. n = 6–7; ** *p* < 0.01, *** *p* < 0.001, **** *p* < 0.0001; one-sample t-test against 0 h phase shift. (**d**) Phase shifts of clock gene expression peaks between day 1 and day 4. Mean activity shifts are indicated by dotted vertical lines. Data are shown as means ± SEM; n = 6–7; ^##^ *p* < 0.01, ^####^ *p* < 0.0001 phase angle difference between feeding regimes; two-way ANOVA with Sidak’s post-tests; * *p* < 0.05, *** *p* < 0.001, **** *p* < 0.0001 phase angle difference between phase shifts of clock gene expression rhythms and mean phase shifts observed for activity onsets within one feeding regime.

On the fourth day—and in line with the lengthened LD (− 28) cycle—gene expression rhythms in all tissues were phase-delayed compared to day 1 (Figs. 2 and 3). In liver, clock gene expression rhythms were shifted by 2.6 ± 0.5 h under LD-28/FF-24 compared to 5.8 ± 0.9 h under LD-28/FF-28 conditions (Fig. 3a). In addition, under LD-28/FF-28 conditions hepatic clock gene expression rhythms were overall dampened (Fig. 3a, right panel). In the adrenal, clock gene rhythms were significantly phase-delayed by 3.4 ± 0.3 h under LD-28/FF-24 and by 5.8 ± 0.2 h LD-28/FF-28 conditions on day 4 (Fig. 3b). The largest FF cycle effects were observed for eWAT. Under LD-28/FF-24 conditions, the average phase shift in clock gene expression rhythms on day 4 was 1.6 ± 0.3 h compared to 7.1 ± 0.2 h under LD-28/FF-28 conditions (Fig. 3c). In addition, clock gene expression rhythms were overall dampened in eWAT under LD-28/FF-28 conditions.

In all tissues, phase shifts between day 1 and day 4 were larger under LD-28/FF-28 compared to LD-28/FF-24 conditions (Figs. 2 and 3). In liver, high variations in the phase shifts for single genes were observed. However, overall phase angle difference of clock gene rhythms were significantly larger under LD-28/FF-28 compared to LD-28/FF-24 on day 4 (Fig. 3d). Clock gene phase angle difference also differed significantly between the two feeding regimes in adrenal and eWAT with larger phase shifts under LD-28/FF-28 conditions (Fig. 3d). In LD-28/FF-24, peripheral tissue clocks phase-shifted significantly less compared to locomotor activity, an indirect measure of SCN clock phase^23^, indicating a desynchronization between central and peripheral clock rhythms (Fig. 3d). Phase shifts in adrenal and eWAT but not liver tissue clocks were significantly larger compared to locomotor activity under LD-28/FF-28 conditions. In all tissues, phase angle difference of single peripheral tissue clocks and locomotor activity were smaller under LD-28/FF-28 compared to LD-28/FF-24 conditions. Thus, synchrony with the SCN was maintained to a higher degree under LD-28/FF-28 conditions (Fig. 3d).

In summary, clock gene expression rhythms in peripheral tissues were phase delayed within 4 days of LD-28 conditions, but the extent of this delay was dependent on the feeding regime. Phase coherence across tissues was disrupted under LD-28/FF-24 while it was maintained to a higher extent under LD-28/FF-28 conditions.

### Within-tissue clock gene programs are disrupted under diverging *zeitgeber* input

**Figure 4:**
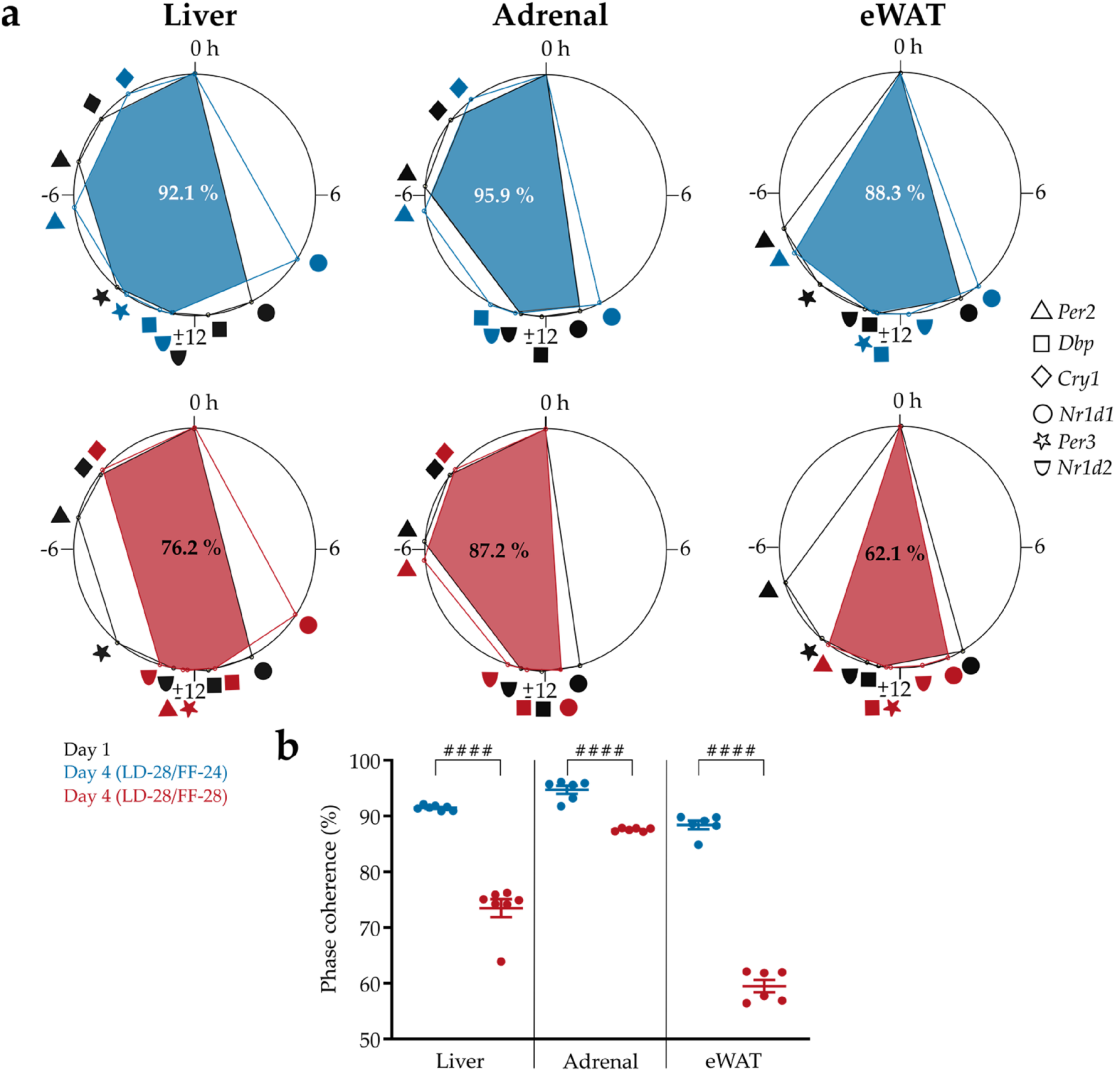
Feeding regimes differentially impact phase coherence within tissues. (**a**) Phase coherence of clock gene expression peaks within tissue on the first (black) and fourth day of the experiment under LD-28/FF-24 (blue) and LD-28/FF-28 (red) conditions. Representative overlaps (coloured areas) are shown for the peak of expression of *Per2* (triangles), *Dbp* (squares), *Cry1* (diamonds), *Nr1d1* (circles), *Per3* (stars), and *Nr1d2* (semicircles) relative to *Bmal1* expression peak (0 h). The area overlaps between day 1 and 4 of the respective FF condition are indicated in the overlapping area. Note, that *Per3* was excluded from further analysis in adrenal since *Per3* was not found to be rhythmic under LD-28/FF-28 conditions. (**b**) Phase coherence calculated from area overlap with each gene serving as reference gene. Data are shown as means ± SEM; n = 6–7; ^###^ *p* < 0.001, ^####^ *p* < 0.0001 between paradigms in the same tissue, two-way ANOVA with Sidak’s post-tests.

To further quantify the impact of the *zeitgeber* food under extended LD cycles on the coordination of clock gene rhythms within single tissues, phase angle differences of individual clock gene rhythms under LD-28/FF-24 and LD-28/FF-28 conditions were compared (Fig. 4a). Phase coherence between the clock genes – *i.e.*, the coordination of peak phases of the different clock genes relative to control (day 1) – within each tissue was decreased on day 4 compared to day 1 for both paradigms. A higher deterioration of phase coherence was observed in every tissue under LD-28/FF-28 conditions (liver: 73.5 ± 1.6%, adrenal: 87.6 ± 0.1%, eWAT: 59.5% ± 1.1% *vs.* liver: 91.5 ± 0.2%, adrenal: 94.7 ± 0.8%, eWAT: 88.4 ± 0.8% for LD-28/FF-24) (Fig. 4b). Of note, phase coherences differed between tissues under LD-28/FF-28 conditions. Under LD-28/FF-24 conditions, only adrenal and eWAT phase coherences were significantly different.

Taken together, within-tissue phase coherence of clock gene rhythms was consistently more affected in LD-28/FF-28 compared to LD-28/FF-24 conditions.

## Discussion

In this study, we investigated the effects of misaligned light–dark (LD) and feeding-fasting (FF) cycles on tissue circadian clock coordination. Mice lengthened their locomotor activity period under LD-28 conditions with little impact of FF period. Across-tissue phase alignment was disrupted under LD-28/FF-24 while it was largely maintained under LD-28/FF-28 conditions. In contrast, within-tissue coherence of clock gene activity, was impaired to a greater extend under LD-28/FF-28 than under LD-28/FF-24 conditions.

Mice were unable to fully entrain to extension of the light–dark cycle to 28 h (LD-28) as seen in previous studies^24–27^. Lengthened activity periods were expected since light is the most potent *zeitgeber* synchronizing the SCN with external time regulating behavioural rhythms^8,28,29^. Phase angle differences were significantly larger under high (300 lx) compared to low-light LD cycles (3 lx during the light phase). Low light intensity, such as the one used in this study, is sufficient to entrain the circadian system in mice under LD-24 conditions^30^ but is less effective in suppressing locomotor activity (light masking)^7,31,32^. Our observations indicate that decreasing the light intensity weakens—but does not abolish—the impact of the *zeitgeber* light on the circadian system. The SCN integrates photic input from the eye and non-photic signals from other brain regions^33–38^ to shape SCN output coordinating rhythms across the whole body^39–43^. Under LD-28/FF-24 and LD-28/FF-28 conditions with 3 lx illuminations during the light phase, locomotor activity onsets were comparable throughout the four days of experiment and, thus, largely independent of the FF schedule. In line with this, increased activity levels were only observed for fasting periods above 18 h probably reflecting food seeking behaviour^44–46^. In consequence, it is reasonable to assume that the locomotor activity observed in our acute *zeitgeber* misalignment paradigm is representative of the period of the SCN clock^23,47^.

The SCN aligns peripheral tissue clocks with external light–dark cycles, *inter alia*, by coordinating behaviours such as sleeping/waking and feeding/fasting. Many peripheral tissue clocks are reported to be strongly impacted by the *zeitgeber* food under normal LD-24 cycles^8,48–50^. However, in modern societies people are often exposed to chronodisruptive environmental conditions. We tried to mimic this situation in mice by exposing them to extended LD cycles combined with FF cycles to study tissue clock adaptation. On day 4 – the day of maximal *zeitgeber* misalignment *–* clock gene expression rhythms in peripheral tissues were phase-delayed compared to the first day of experiment independent of the imposed feeding schedule. However, the magnitude of this delay was dependent on the FF cycle period. This indicates that feeding-regulated signals such as leptin, ghrelin, insulin, but also glucocorticoids may impact tissue clock resetting. The daily glucocorticoid (GC) rhythm is dependent on SCN signalling^51,52^ but is also regulated by the adrenal cortical clock^22,53^. Activated glucocorticoid receptors induce target gene transcription through glucocorticoid response elements (*GRE*s), which are also found in the *Per2* locus^54–56^. GC and SCN rhythms dissociate under misaligned *zeitgeber* conditions (LD-28/FF-24)^24^, thus weakening the effect of SCN-mediated synchronisation in peripheral tissues. Under LD-28/FF-28 conditions, *zeitgeber* input phase-shifted with the same kinetics. Therefore, a more uniform phase-adaptation of SCN-driven and GC rhythms may provide a more coherent clock resetting stimulus to other tissues, resulting in larger clock gene phase angle differences (see Fig. 3). The mechanisms how food-related signals may reset peripheral tissue clocks are still poorly understood, but it is plausible that various signals are integrated to generate and coordinate clock oscillations across tissues. Some of the best studied food-related signals which modulate tissue clocks are the counteracting adipose tissue-derived hormone leptin and gut-derived hormone ghrelin which signal the organism’s metabolic state to the brain^57^. Leptin and ghrelin can restore clock gene expression in vitro and in vivo in obese animal models^58–60^. Insulin, released by pancreatic β-cells, can modulate the expression and translation of various clock genes including *Per2*, *Rev-Erb α*, *Clock* and *Bmal1*^61–63^. Additionally, the gut-derived hormone oxyntomodulin may affect clock resetting by activating the expression of *Per1* and *Per2*^64^. There are many more food-related signals and metabolites some of which may affect tissue clock regulation (reviewed in^65^) making it difficult to unravel their differential contributions on clock resetting in *in-vivo* experiments. Under disruptive *zeitgeber* input numerous systemic signals are likely impacted which in turn differentially influence tissue clock resetting. The observed phase shifts for the adrenal clock under LD-28/FF-24 and LD-28/FF-28 conditions emphasize that the adrenal is susceptible to resetting signals of both *zeitgebers*, light and food^66–68^. Liver clocks are known to be exceptionally susceptible to food-related resetting signals, but also glucocorticoid signalling^8,9,48^. Surprisingly, FF schedules had the strongest impact on eWAT. Adipose tissue receives various (oscillating) signals, *e.g.* glucocorticoids, glucose and insulin which impact adipose tissue physiology (reviewed in^69^). A limitation of this study is that clock gene tissue profiles were obtained at relatively low temporal resolution which may impact the accuracy of determining the time of peak expression. Higher sampling rates may further improve the precision of peak time determination. However, calculations using previously published short-interval data sets^22,70^ suggest only minor (< 0.5 h) effects of sampling rate on peak time accuracy between 3-h and 8-h sampling intervals.

Under LD-28/FF-24 conditions, phase shifts of all peripheral tissue clocks were smaller than the phase shift seen for locomotor activity period. In contrast, adrenal and eWAT but not liver showed a significantly larger phase shift compared to the locomotor activity phase shift under LD-28/FF-28 conditions. These results indicate that internal desynchrony – *i.e.,* phase misalignment between tissues – was induced in the LD-28/FF-24 paradigm whereas a higher degree of synchrony was maintained in the LD-28/FF-28 paradigm. Regarding within-tissue coherence, however, LD-28/FF-24 worked better than LD-28/FF-28 indicating a higher stability of circadian organization at the cellular level. Physiological functions may be affected at both levels of organisation which makes it difficult to make recommendations for stabilizing circadian alignment under disruptive *zeitgeber* conditions such as shift work or transcontinental travel. On one hand, it might be advisable to change *zeitgeber* input with the same kinetics—*e.g.*, by aligning food intake rhythms to rotating shift schedules. On the other hand, within tissue clock rhythms may be stabilized by keeping the feeding regime aligned with the 24-h cycle, thus promoting circadian coherence at the cellular level. Future experiments are needed to investigate the underlying mechanisms and physiological consequences.

Together, we here show for the first time that circadian clock phase alignment is reduced across- but maintained to a higher degree within-tissue under LD-28/FF-24 conditions with opposite effects under LD-28/FF-28 conditions. Both paradigms mimic chronodisruptive environmental conditions as may be experienced during shift work. Shift workers show alterations in their sleep/wake behaviour^71,72^ and meal timing relative to the LD cycle (reviewed in^73,74^) which is assumed to reflect internal circadian misalignment and favour the development of metabolic and cardiovascular disorders. It remains to be shown if circadian misalignment at systemic or within-tissue levels underlies these adverse health outcomes. The protocols established here provide a tool for future experiments in this direction.

## Methods

### Animals

Young adult male C57BL/6 J mice, 10–17 weeks old, were maintained in the animal facility of the University of Lübeck. *Prior* to the experiments mice were acclimatized for 1 week to single-housing and running-wheel cages under 12-h light: 12-h dark conditions (LD-24; Fig. 1a). Illumination was set to 300 lx during the light phase. Animals had ad libitum access to chow food (Altromin #1314) and water. After acclimatisation, mice were released into 14-h light: 14-h darkness (LD-28) conditions and illumination during the light phases was left unchanged (300 lx) or reduced to 3 lx. Food access was either restricted to 12-h feeding: 12-h fasting (FF-24, with food access coinciding with the LD-24 dark phase) or to 14-h feeding: 14-h fasting (FF-28, with food access coinciding with the LD-28 dark phase). Chow was removed 1 h after "lights on"/ZT1 (LD-28/FF-24, 300 lx and 3 lx) or at "lights on"/ZT0 (LD-28/FF-28) on day 1. All animal experiments were designed in accordance with the German Law for Animal Protection (TierSchG), ethically assessed and legally approved by the ethics commission of the Ministry of Energy Transition, Agriculture, Environment, Nature and Digitalization (MELUND) of the State of Schleswig–Holstein, Germany, and reported in accordance with the ARRIVE guidelines.

### Behavioural measurements

Running-wheel activity of mice was recorded and analysed using the ClockLab system and software (6.0.34, Actimetrics, Evanston, USA). Entrainment of mice to LD-24 conditions and running-wheel usage were ensured by activity analysis during acclimatisation. Mice were accustomed to the noise created by emptying and refilling the food hoppers for several days before the start of the experiment.

### Tissue and serum collections

On the first day and fourth day of LD-28 animals were sacrificed at evenly spaced time points (every 7 h LD-28/FF-28 or 6 h for LD-28/FF-24) spanning the 28-h LD cycle. The first day of LD-28/FF-28 served as baseline profile (day 1) for both feeding regimes. Mice were sacrificed by cervical dislocation followed by immediate decapitation. In the dark phase, sacrificing was performed under dim red light followed by removal of the eyes before turning on the lights for tissue dissection. Tissues were stored in RNA*later* (ThermoFisher, Waltham, USA) at − 20 °C before RNA isolation.

### RNA isolation and quantitative real-time (q)PCR

RNA isolation was performed as described previously^24^. Briefly, tissues were homogenized, and total RNA was extracted using TRIzol reagent (ThermoFisher). RNA was transcribed into cDNA using random-hexamer primers and High-capacity cDNA Reverse Transcription Kit (Applied Biosystems, Foster City, USA) following the manufacturer’s protocol. cDNAs were diluted 1:10–1:20 and stored at − 20 °C. qPCR was done using Go-Taq qPCR Master Mix (Promega, Madison, USA) on a Bio-Rad CFX96 thermocycler (Bio-Rad, Hercules, USA). The following primers were used: *Eef1a* forward 5’-TGCCCCAGGACACAGAGACTTCA-3’; *Eef1a* reverse 5’-AATTCACCAACACCAGCAGCAA-3’; *Bmal1* forward 5’CCTAATTCTCAGGGCAGCAGAT-3’; *Bmal1* reverse 5’-TCCAGTCTTGGCATCAATGAGT-3’; *Per2* forward 5’- GCCAAGTTTGTGGAGATTCCTG-3’; *Per2* reverse 5’-CTTGCACCTTGACCAGGTAGG-3’; *Dbp* forward 5’-AATGACCTTTGAACCTTGATCCCGCT-3’; *Dbp* reverse 5’-GCTCCAGTACTTCTCATCCTTCTGT-3’; *Nr1d1* forward 5’-AGCTCAACTCCCTGGCACTTAC-3’; *Nr1d1* reverse 5’- CTTCTCGGAATGCATGTTGTTC-3’; *Cry1* forward 5’-GTCATTGCAGGAAAATGGGAAG-3*’; Cry1* reverse 5’- TAAAGAGGCGGAGAGACAAAGG-3’*; Per3* forward 5’- GTGACAGCAGAGTCCCATGA-3’*; Per3* reverse 5’- CACTGCCATCTCGAGTTCAA-3’; *Nr1d2* forward 5’- TCATGAGGATGAACAGGAACC-3’; *Nr1d2* reverse 5’- GAATTCGGCCAAATCGAAC-3’.

### Data analyses and statistics

Activity profiles were generated from 20-min bins of individual running-wheel activity extracted from ClockLab data^24^. Daily averages of total activity were calculated for four days of LD-24 and used for computing relative activity per bin size for experimental days. Activity profiles were smoothed using 3-neighbour running averages with GraphPad Prism 7 (GraphPad Software, San Diego, USA). Activity onsets were determined by visual inspection on actogram plots. Activity period (τ) in LD-24 and LD-28 conditions was determined by fitting a straight line to activity onsets over at least three days. Statistical differences in activity were analysed by one-way ANOVA with Tukey’s multiple comparisons tests. The average total phase shift of activity was calculated from the fits which served as a reference for determining statistical differences between central and peripheral tissue clock outputs.

mRNA expression levels were normalised to *Eukaryotic elongation factor-1 α* (*Eef1a*). Relative expression ratios (using the ΔΔCT method) were calculated^13^ and normalized to the mean ratio of the profile on day 1. Outliers were detected by Dean Dixon test with α = 0.01 and excluded from further analysis. Rhythmicity of diurnal gene expression profiles was tested using CircWave 1.4^75^ and non-rhythmic genes were excluded from further analysis. Amplitude changes were tested for significance by one-sample t-tests against the hypothetical value of 100% (*i.e.*, the mean of day 1 expression ratios). Maxima of gene expression were calculated from sine wave fits with a fixed wavelength of 25.8 h (GraphPad), the average behavioural period length under all LD-28 conditions (Figure S1). Phase shifts of maximum gene expression were calculated between day 1 and 4 under the different FF regimes. Phase shifts of gene expression were tested against the hypothetical value of 0 (corresponds to gene expression maxima on day 1) in one-sample t-tests and plotted in Oriana, version 4 (Kovach Computing Services, Anglesey, United Kingdom). Statistical differences in phase angle between the two intervention paradigms (LD-28/FF-24 *vs.* LD-28/FF-28) were tested by two-way ANOVA with Sidak's post-tests. Phase shifts of peripheral gene expression were tested against the mean phase shift of locomotor activity using one-sample t-tests. For the assessment of phase coherence of clock gene expression rhythms within a specific tissue, sine fit expression peaks of a reference clock gene were set to 0 h and expression peaks of the other clock genes were plotted relative to the reference clock gene. This procedure was repeated for all clock genes. Subsequently, the overlapping areas of the obtained polygons of day 1 and the respective day 4 (LD-28/FF-24 or LD-28/FF-28) were calculated using SketchAndCalc (www.sketchandcalc.com). To test if feeding regime has an impact on phase coherence in the different tissues, overlapping areas were tested in a two-way ANOVA with Sidak’s post-tests. In all analyses,* p* values below 0.05 were considered significant.

## Supplementary Information

Supplementary Information.
