# Supplementary material for: Induction of internal circadian desynchrony by misaligning zeitgebers
Source: Sci Rep. 2022 Jan 31;12:1601. doi: 10.1038/s41598-022-05624-x (PMC8803932; doi:10.1038/s41598-022-05624-x)
Supplement: Supplementary file 1 — Supplementary Information. [file 41598_2022_5624_MOESM1_ESM.docx]

**Supplements**

**Title**

Induction of internal circadian desynchrony by misaligning *zeitgebers*

**Authors**

Isabel Heyde^1^ & Henrik Oster^1.^*

**Affiliations**

^1^: Institute of Neurobiology, University of Lübeck, Germany

**Contact**

Henrik Oster, University of Lübeck, Institute of Neurobiology, CBBM (House 66), Marie Curie Street, 23562 Lübeck, Germany, Email: henrik.oster@uni-luebeck.de, Phone: +49 451 3101 4300


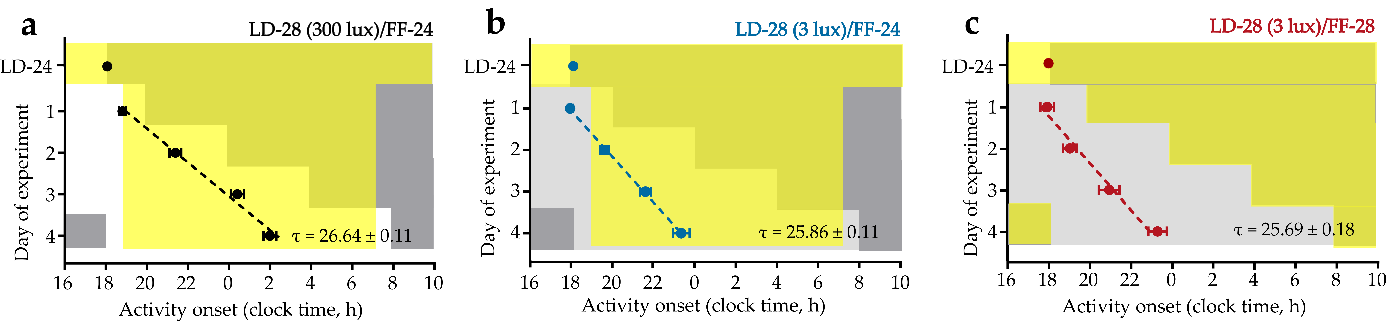


**Supplemental figure 1.** Daily activity onsets under (a) LD-28/FF-24 (300 lux, n=46-55), (b) LD-28/FF-24 (3 lux, n=58-66) and (c) LD-28/FF-28 (3 lux, n=8-15) conditions. Dark phases and food access times are indicated in dark grey and yellow shadings, respectively. Light grey shading in (b, c) indicate experimental light phase (3 lux). Data are shown as means ± SEM.


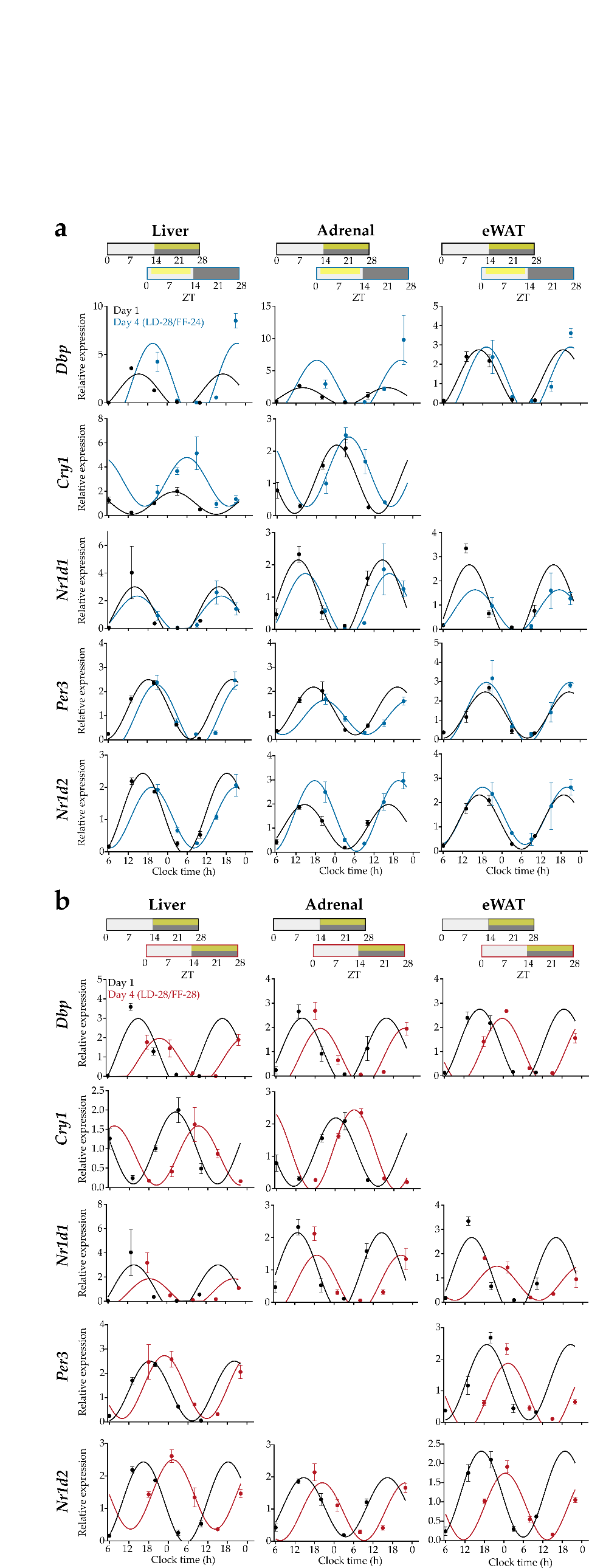


**Supplemental figure 2.** Clock gene expression profiles on the first and fourth day under LD-28/FF-24 and LD-28/FF-28 conditions. Diurnal mRNA expression profiles over 28 h for *Dbp*, *Cry1,* *Nr1d1*, *Per3* and *Nr1d2* (top to bottom) on the first day of experiment (a, b) under LD-28/FF-28 conditions (black) and fourth day of experiment under (a) LD-28/FF-24 (blue) and (b) LD-28/FF-28 (red) conditions in liver, adrenal and eWAT (left to right). Data are shown as means ± SEM, n=3-5 animals per time point. Fitted curves are sine waves with a wavelength of 25.8 h. Note, that diurnal expression profiles which were not rhythmic on the fourth day of experiment under LD­-28/FF-24 and LD-28/FF-28 conditions were excluded from further analysis and are, therefore, not shown. Light grey, dark grey and yellow bars on top indicate light phase, dark phase and food access time on the corresponding day.
